# Composition of Physiology

**Published:** 1896-06

**Authors:** 


### Composition on Physiology.

The following composition by a twelve-year-old school boy was the cause of his being recommended to take a special course in physiology next term. The theme given him was "breath."

"Breath is made of air. We always breathe with our lungs, and sometimes with our livers, except at night, when our breath keeps life going through our noses while we are asleep. If it wasn't for our breath we should die whenever we slept. Boys that stay in a room all day should not breathe; they should wait till they get outdoors. For a lot of boys staying in a room make carbonicide, and carbonicide is more poisonous than mad dogs, though not just the same way. It does not bite; but that's no matter as long as it kills you."—*British Medico-Chirurgical Journal.*

